# Supplementary material for: Modeling glioblastoma heterogeneity as a dynamic network of cell states
Source: Mol Syst Biol. 2021 Sep 16;17(9):e10105. doi: 10.15252/msb.202010105 (PMC8444284; doi:10.15252/msb.202010105)
Supplement: Supplementary file 6 — Source Data for Figure 5 [file MSB-17-e10105-s004.zip › Figure5A_sourcedata/GSEA_3071/nosigdb_stateIV.GseaPreranked.1622795164626/LARSSON_STATE_4.html]

Details for gene set LARSSON\_STATE\_4[GSEA]

|  || Dataset | state53071 |
| Phenotype | NoPhenotypeAvailable |
| Upregulated in class | na\_neg |
| GeneSet | LARSSON\_STATE\_4 |
| Enrichment Score (ES) | -0.75678134 |
| Normalized Enrichment Score (NES) | -3.887141 |
| Nominal p-value | 0.0 |
| FDR q-value | 0.0 |
| FWER p-Value | 0.0 |
Table: GSEA Results Summary

  

Fig 1: Enrichment plot: LARSSON\_STATE\_4      
 Profile of the Running ES Score & Positions of GeneSet Members on the Rank Ordered List

  

| PROBE | GENE SYMBOL | GENE\_TITLE | RANK IN GENE LIST | RANK METRIC SCORE | RUNNING ES | CORE ENRICHMENT || 1 | TMSB15A |  |  | 1540 | 0.381 | -0.2911 | No |
| 2 | PTTG1 |  |  | 2470 | -0.266 | -0.4663 | No |
| 3 | LMNB1 |  |  | 2487 | -0.268 | -0.4660 | No |
| 4 | HMGB1 |  |  | 2819 | -0.308 | -0.5257 | No |
| 5 | EZH2 |  |  | 2946 | -0.324 | -0.5458 | No |
| 6 | SMC4 |  |  | 3035 | -0.337 | -0.5585 | No |
| 7 | TUBA1B |  |  | 3357 | -0.383 | -0.6153 | No |
| 8 | CENPF |  |  | 4003 | -0.511 | -0.7328 | No |
| 9 | HIST1H1E |  |  | 4011 | -0.515 | -0.7277 | No |
| 10 | PHF19 |  |  | 4041 | -0.523 | -0.7266 | No |
| 11 | TUBB |  |  | 4083 | -0.534 | -0.7278 | No |
| 12 | ANP32B |  |  | 4235 | -0.576 | -0.7495 | Yes |
| 13 | HMGB2 |  |  | 4243 | -0.578 | -0.7436 | Yes |
| 14 | SNRPD1 |  |  | 4254 | -0.582 | -0.7382 | Yes |
| 15 | SIVA1 |  |  | 4263 | -0.586 | -0.7323 | Yes |
| 16 | KIF15 |  |  | 4324 | -0.603 | -0.7362 | Yes |
| 17 | ASF1B |  |  | 4328 | -0.606 | -0.7291 | Yes |
| 18 | SGO1 |  |  | 4339 | -0.611 | -0.7234 | Yes |
| 19 | KPNA2 |  |  | 4361 | -0.621 | -0.7196 | Yes |
| 20 | ALYREF |  |  | 4406 | -0.638 | -0.7200 | Yes |
| 21 | RPA3 |  |  | 4482 | -0.670 | -0.7259 | Yes |
| 22 | H2AFX |  |  | 4495 | -0.677 | -0.7197 | Yes |
| 23 | DHFR |  |  | 4517 | -0.688 | -0.7150 | Yes |
| 24 | MND1 |  |  | 4538 | -0.699 | -0.7101 | Yes |
| 25 | DTYMK |  |  | 4566 | -0.715 | -0.7062 | Yes |
| 26 | NCAPG |  |  | 4568 | -0.716 | -0.6974 | Yes |
| 27 | DBF4 |  |  | 4570 | -0.717 | -0.6885 | Yes |
| 28 | RFC2 |  |  | 4588 | -0.729 | -0.6826 | Yes |
| 29 | PBK |  |  | 4593 | -0.730 | -0.6742 | Yes |
| 30 | CKS2 |  |  | 4600 | -0.733 | -0.6661 | Yes |
| 31 | BIRC5 |  |  | 4618 | -0.745 | -0.6599 | Yes |
| 32 | PTMA |  |  | 4620 | -0.746 | -0.6507 | Yes |
| 33 | E2F1 |  |  | 4637 | -0.760 | -0.6442 | Yes |
| 34 | DUT |  |  | 4642 | -0.762 | -0.6354 | Yes |
| 35 | SGO2 |  |  | 4650 | -0.764 | -0.6271 | Yes |
| 36 | NUSAP1 |  |  | 4654 | -0.765 | -0.6180 | Yes |
| 37 | FANCI |  |  | 4665 | -0.772 | -0.6102 | Yes |
| 38 | CENPH |  |  | 4679 | -0.786 | -0.6028 | Yes |
| 39 | CDC45 |  |  | 4702 | -0.798 | -0.5969 | Yes |
| 40 | MCM7 |  |  | 4704 | -0.800 | -0.5870 | Yes |
| 41 | GTSE1 |  |  | 4722 | -0.808 | -0.5801 | Yes |
| 42 | KIF4A |  |  | 4727 | -0.812 | -0.5706 | Yes |
| 43 | RANBP1 |  |  | 4741 | -0.823 | -0.5627 | Yes |
| 44 | RAN |  |  | 4743 | -0.824 | -0.5525 | Yes |
| 45 | CHAF1A |  |  | 4751 | -0.828 | -0.5434 | Yes |
| 46 | TMPO |  |  | 4790 | -0.851 | -0.5400 | Yes |
| 47 | MCM5 |  |  | 4792 | -0.853 | -0.5294 | Yes |
| 48 | CENPN |  |  | 4796 | -0.858 | -0.5191 | Yes |
| 49 | MCM3 |  |  | 4834 | -0.901 | -0.5149 | Yes |
| 50 | MKI67 |  |  | 4836 | -0.902 | -0.5037 | Yes |
| 51 | SMC2 |  |  | 4847 | -0.912 | -0.4941 | Yes |
| 52 | UHRF1 |  |  | 4859 | -0.924 | -0.4846 | Yes |
| 53 | ORC6 |  |  | 4865 | -0.931 | -0.4738 | Yes |
| 54 | GMNN |  |  | 4873 | -0.939 | -0.4633 | Yes |
| 55 | HIST1H1C |  |  | 4877 | -0.940 | -0.4520 | Yes |
| 56 | CENPM |  |  | 4881 | -0.947 | -0.4406 | Yes |
| 57 | GINS2 |  |  | 4897 | -0.962 | -0.4314 | Yes |
| 58 | FBXO5 |  |  | 4910 | -0.978 | -0.4213 | Yes |
| 59 | H2AFZ |  |  | 4919 | -0.983 | -0.4105 | Yes |
| 60 | BRCA1 |  |  | 4938 | -1.002 | -0.4013 | Yes |
| 61 | MCM4 |  |  | 4947 | -1.011 | -0.3900 | Yes |
| 62 | CCNB1 |  |  | 4962 | -1.032 | -0.3797 | Yes |
| 63 | ATAD2 |  |  | 4980 | -1.058 | -0.3696 | Yes |
| 64 | RRM1 |  |  | 4990 | -1.074 | -0.3578 | Yes |
| 65 | UBE2T |  |  | 5000 | -1.091 | -0.3458 | Yes |
| 66 | MAD2L1 |  |  | 5007 | -1.105 | -0.3330 | Yes |
| 67 | TK1 |  |  | 5020 | -1.126 | -0.3211 | Yes |
| 68 | TOP2A |  |  | 5029 | -1.152 | -0.3081 | Yes |
| 69 | MYBL2 |  |  | 5041 | -1.172 | -0.2954 | Yes |
| 70 | TPX2 |  |  | 5049 | -1.184 | -0.2818 | Yes |
| 71 | PCNA |  |  | 5060 | -1.214 | -0.2684 | Yes |
| 72 | MCM10 |  |  | 5066 | -1.222 | -0.2540 | Yes |
| 73 | ESCO2 |  |  | 5067 | -1.222 | -0.2386 | Yes |
| 74 | CENPK |  |  | 5091 | -1.267 | -0.2270 | Yes |
| 75 | UBE2C |  |  | 5106 | -1.314 | -0.2131 | Yes |
| 76 | ZWINT |  |  | 5115 | -1.321 | -0.1980 | Yes |
| 77 | CKS1B |  |  | 5121 | -1.338 | -0.1820 | Yes |
| 78 | RAD51AP1 |  |  | 5133 | -1.375 | -0.1668 | Yes |
| 79 | TYMS |  |  | 5156 | -1.477 | -0.1524 | Yes |
| 80 | CENPU |  |  | 5159 | -1.486 | -0.1340 | Yes |
| 81 | CDK1 |  |  | 5164 | -1.512 | -0.1157 | Yes |
| 82 | CLSPN |  |  | 5202 | -1.663 | -0.1019 | Yes |
| 83 | PCLAF |  |  | 5209 | -1.698 | -0.0816 | Yes |
| 84 | HIST1H1B |  |  | 5223 | -1.811 | -0.0612 | Yes |
| 85 | HIST1H1D |  |  | 5231 | -1.871 | -0.0390 | Yes |
| 86 | RRM2 |  |  | 5232 | -1.875 | -0.0153 | Yes |
| 87 | HIST1H4C |  |  | 5249 | -2.081 | 0.0079 | Yes |
Table: GSEA details [plain text format]

  

Fig 2: LARSSON\_STATE\_4: Random ES distribution      
 Gene set null distribution of ES for **LARSSON\_STATE\_4**
